# Maternal Morbidity With Expectant Management of Life-Limiting Fetal Conditions

**DOI:** 10.1001/jamanetworkopen.2025.21883

**Published:** 2025-07-18

**Authors:** Anjali Nambiar, Elaine Larissa Duryea, Lisa Renee Thiele, Patricia Santiago-Munoz, David B. Nelson, Catherine Y. Spong, Courtney C. Baker

**Affiliations:** 1 Department of Obstetrics and Gynecology, University of Texas Southwestern Medical Center, Dallas; 2 Parkland Health, Dallas, Texas

## Abstract

This cohort study assesses maternal morbidity in pregnancies with life-limiting fetal conditions before and after management options were limited by state legislation.

## Introduction

Pregnancy management following diagnosis of a life-limiting fetal condition can be emotionally and medically complex. Comprehensive counseling and shared decision-making allow patients to choose health care options that balance their values and safety, including decisions regarding pregnancy duration.^1,2^ Pregnancy continuation resulting from expectant management may expose patients to pregnancy-associated complications (eg, hemorrhage, infection, hypertension); however, limited data exist to aid decision-making. We report perinatal outcomes from a single institution serving primarily uninsured or Medicaid-insured patients in pregnancies with life-limiting fetal conditions before and after management options were limited by state legislation.

## Methods

This cohort study included patients with a singleton pregnancy diagnosed at less than 22 weeks’ gestation with a life-limiting fetal condition before changes in state legislation (January 2017 to December 2018) and after (January 2022 to December 2023). The University of Texas Southwestern Medical Center Institutional Review Board deemed this study of minimal risk to patients and waived informed consent. This study followed the STROBE reporting guideline.

Before 2021, our institution offered expectant management or previable termination of pregnancy for life-limiting fetal conditions, including trisomy 13 and 18, in which postnatal life expectancy is generally extremely limited, and major structural anomalies (bilateral renal agenesis or multicystic or dysplastic kidneys with anhydramnios, severe skeletal dysplasia, alobar holoprosencephaly, anencephaly, body stalk anomaly), in which death occurs shortly following delivery at any gestational age regardless of medical intervention. After state legislation changes, in the absence of a maternal life-threatening condition, only expectant management was offered.^3^ Maternal morbidity and neonatal outcomes (stillbirth, infant or neonatal death) were examined. Composite maternal morbidity included chorioamnionitis, endometritis, hysterotomy, hemorrhage, transfusion, preeclampsia, abruption, death, and postpartum readmission. Race and ethnicity data were not collected to minimize reporting of patient-identifying information and protect patient privacy. Patients who underwent planned surgery for placenta accreta spectrum, were lost to follow-up, or received treatment outside our facility were excluded from morbidity analysis.

Obstetric outcomes and demographics were evaluated using χ^2 ^and *t* tests, respectively. Morbidity analysis was performed using Fisher exact test. Significance was considered at *P* ≤ .05. The analyses were performed using SAS, version 9.4 (SAS Institute Inc).

## Results

Before legislation changes, 53 patients (mean [SD] maternal age, 31 [7] years) were diagnosed at a mean (SD) of 17.7 (2.4) weeks’ gestation, of whom 29 (55%) chose to terminate pregnancy and 24 (45%) chose expectant management (Table 1). After legislation, 34 patients (mean [SD] maternal age, 32 [8] years) were diagnosed at a mean (SD) of 18.4 (2.0) weeks, of whom 4 were lost to follow-up. Of the 30 remaining patients, 26 (87%) underwent expectant management and 4 (13%) terminated their pregnancy at an out-of-state facility (Table 2). All pregnancies undergoing expectant management resulted in stillbirth or neonatal or infant death, with a nonsignificant increase in infant or neonatal death rate after legislation (46% vs 69%; *P* = .09). Incidence of preeclampsia and cesarean delivery increased after legislation and occurred only in patients treated with expectant management. The composite maternal morbidity rate following expectant management was significantly higher vs pregnancy termination (72% vs 15%; *P* < .001) and between cohorts before vs after legislation changes (35% vs 72%; *P* = .003). Post hoc analysis showed that significance was driven by the difference in the pregnancy termination vs expectant management cohorts.

**Table 1.** Maternal Demographics for Pregnancies With Life-Limiting Fetal Conditions, Before and After State Legislation Changes

Demographic	Mean (SD)		*P* value
	Before (n = 53)	After (n = 34)	
Maternal age, y	31 (7)	32 (8)	.31
Gravida	3 (2)	4 (2)	.46
Parity	2 (2)	2 (1)	.38
Nulliparity, No. (%)	12 (23)	7 (21)	.82
EGA at diagnosis, wk	17.7 (2.4)	18.4 (2.0)	.10

Abbreviation: EGA, estimated gestational age.

**Table 2.** Obstetric Outcomes and Morbidity for Pregnancies With Life-Limiting Conditions, Before and After State Legislation Changes

Outcome	No./total No. in analysis (%)			*P* value
	Before (n = 53)		After (n = 30)^a^	
	Termination of pregnancy	Expectant management	Expectant management	
**Obstetric**				
No. of patients	29	24	30	NA
EGA at termination of pregnancy, mean (SD), wk	18 (2.5)	NA	NA	NA
EGA at delivery, mean (SD), wk^b^	NA	33 (6.9)	34 (5.2)	NA
Outside facility termination of pregnancy	1/53 (2)	NA	4/30 (13)	.04
Outside facility delivery	NA	4/24 (17)	1/26 (4)	.13
Vaginal delivery (expectant management)	NA	16/24 (67)	15/26 (58)	.51
Cesarean delivery (expectant management)^c^	NA	8/24 (33)	11/26 (42)	.51
**Fetal and neonatal **				
Stillbirth	NA	13/24 (54)	8/26 (31)	.09
Infant or neonatal death	NA	11/24 (46)	18/26 (69)	.09
Postnatal survival, median (IQR), h	NA	17 (5-216)	3 (1-96)	NA
**Maternal morbidity analysis^d^**				
No. of patients	26	20	25	NA
Hemorrhage^e^	1/26 (4)	6/20 (30)	9/25 (36)	.009
Transfusion	0	2/20 (10)	3/25 (12)	.21
Chorioamnionitis^f^	3/25 (12)	4/19 (21)	4/22 (18)	.71
Endometritis	0	2/20 (10)	0	.08
Hysterotomy or laparotomy	0	8/20 (40)	10/25 (40)	<.001
Preeclampsia	0	3/20 (15)	6/25 (24)	.02
Abruption	0	2/20 (10)	1/25 (4)	.19
Postpartum readmission	0	1/20 (5)	1/25 (4)	.53
Maternal death	0	0	0	NA
Composite morbidity^g^	4/26 (15)	12/20 (60)	18/25 (72)	<.001

Abbreviations: EGA, estimated gestational age; NA, not applicable.

^a^ Number of patients after state legislation change was 30 due to 4 patients lost to follow-up. These patients were not included in subsequent analyses.

^b^ Includes EGA at delivery of stillbirth or live neonate.

^c^ Overall cesarean delivery more than doubled between the 2 cohorts from 8 of 53 (15%) before the legislative change to 11 of 30 (37%) after the change (*P* = .03).

^d^ Excluded from morbidity analysis as patients either underwent planned surgery for placenta accreta spectrum or termination of pregnancy or delivery at an outside facility.

^e^ Defined as blood loss of greater than 500 mL for vaginal delivery or termination of pregnancy and greater than 1000 mL for cesarean delivery.

^f^ Defined as chorioamnionitis on placenta pathology. Pathology unavailable for 5 patients. Of note, the majority of pregnancy terminations were performed via medical induction of labor.

^g^ The combined composite morbidity doubled between the 2 cohorts from 16 of 46 patients (35%) before the legislative change to 18 of 25 patients (72%) after the change (*P* = .003).

## Discussion

This cohort study shows that universal expectant management of life-limiting fetal conditions resulting from legislation changes was associated with significantly higher maternal morbidity, similar to outcomes in the setting of previable rupture of membranes.^4^ More infant or neonatal deaths were observed following legislation changes, consistent with public health data analyses^5^ and possibly due to higher rates of expectant management. Study limitations included the retrospective nature of data collection, small sample size due to rare prenatal diagnoses, and limited morbidity analysis for patients lost to follow-up or who received care outside our institution. Further research is warranted to examine the impact of reproductive legislation on subsequent pregnancies and long-term health outcomes for this population.

## Notes

Supplement.
Data Sharing Statement
