# Error in Affiliations

**DOI:** 10.1001/jamanetworkopen.2025.30369

**Published:** 2025-08-13

In the Research Letter titled “Maternal Morbidity With Expectant Management of Life-Limiting Fetal Conditions,”^1^ published July 18, 2025, there was an error in the affiliations for all authors. A secondary affiliation, Parkland Health, Dallas, Texas, was added for all authors. This article has been corrected.^1^
