# Supplementary material for: Maternal Morbidity With Expectant Management of Life-Limiting Fetal Conditions
Source: JAMA Netw Open. 2025 Jul 18;8(7):e2521883. doi: 10.1001/jamanetworkopen.2025.21883 (PMC12274976; doi:10.1001/jamanetworkopen.2025.21883)
Supplement: Supplement. — Data Sharing Statement [file jamanetwopen-e2521883-s001.pdf]

Data Sharing Statement

Nambiar. Maternal Morbidity With Expectant Management of Life-Limiting Fetal Conditions. *JAMA Netw Open*. Published July 18, 2025. doi:10.1001/jamanetworkopen.2025.21883

Data
Data available: No
